# External Counterpulsation Attenuates Hypertensive Vascular Injury Through Enhancing the Function of Endothelial Progenitor Cells

**DOI:** 10.3389/fphys.2020.590585

**Published:** 2021-02-12

**Authors:** Jianwen Liang, Jian Shi, Wenbin Wei, Guifu Wu

**Affiliations:** ^1^Department of Cardiology, The Eighth Affiliated Hospital of Sun Yat-sen University, Shenzhen, China; ^2^Department of Cardiology, The Third Affiliated Hospital of Sun Yat-sen University, Shenzhen, China; ^3^Guangdong Innovative Engineering and Technology Research Center for Assisted Circulation, Shenzhen, China

**Keywords:** hypertension, external counterpulsation, vascular injury, endothelial progenitor cells, treatment

## Abstract

**Background:**

Vascular injury is a landmark of hypertension and enhanced external counterpulsation (EECP) has been identified as a noninvasive treatment to restore the capacity of endothelial cells. However, the effect of EECP on blood pressure lowering in hypertension and the potential mechanism remain unknown.

**Methods:**

We measured the ambulatory blood pressure (AMBP) and flow-mediated endothelial dilation (FMD) in the essential hypertensive patients who were randomly assigned to the EECP group (*n* = 20) or control group (*n* = 20). We also evaluated *in vitro* function of endothelial progenitor cells (EPCs). Furthermore, multivariate analysis was performed to determine the actual correlation between EPC function and FMD.

**Results:**

Compared with the control, EECP group exhibited decreased systolic [(133.2 ± 4.9) mmHg vs. (139.3 ± 6.4) mmHg, *P* < 0.05] and diastolic [(83.4 ± 4.5) mmHg vs. (89.5 ± 7.6) mmHg, *P* < 0.05] blood pressure and increased FMD value [(8.87 ± 2.46%) vs. (7.51 ± 2.32%), *P* < 0.01]. In addition, the migration [(47.3 ± 6.4)/hpf vs. (33.4 ± 5.1) hpf, *P* < 0.05] and adhesion [(45.1 ± 5.5)/hpf vs. (28.4 ± 3.9) hpf, *P* < 0.05] functions of EPCs in the EECP group were improved significantly, whereas no change was observed in the control. Both migration [odds ratio (OR) = 0.47, 95% confidence interval (CI) = 0.27–0.64, *P* < 0.05] and adhesion (OR = 0.44, 95% CI = −0.0034 to 0.0012, *P* < 0.05) of EPCs correlated with FMD. After multivariate analysis, the migration (*β* = 3.37, 95% CI = 1.67–5.33, *P* < 0.05) and adhesion (*β* = 3.98, 95% CI = 1.12–6.43, *P* < 0.05) functions still independently correlated to FMD.

**Conclusion:**

The present study demonstrates for the first time that EECP decreases both systolic and diastolic blood pressure and increases FMD value in hypertension. The fall in endogenous EPCs repair capacity might be an important mechanism of hypertensive vascular injury and could be restored by EECP.

## Introduction

Hypertension affects 30% of Chinese adult population and remains the most important risk factor for cardiovascular disease (Forouzanfar et al., 2017). Vascular injury is the landmark of pathophysiological change of hypertension and results in endothelial cell adhesion molecule expression, leukocyte recruitment, macrophage lipid accumulation, and foam cell formation (Pothineni et al., 2017; Zhong et al., 2018). Therefore, it would be of great significance to develop therapeutic methods not only to lower blood pressure (BP) but also to repair vascular injury.

Accelerated re-endothelialization is an important approach for injured artery repair. Our previous study demonstrated that circulating endothelial progenitor cell (EPC) number and activities declined in hypertension (Luo et al., 2016). Accumulating evidence suggests that EPCs provide an endogenous repair mechanism to counteract ongoing risk factor–induced endothelial injury and to replace impaired endothelium (Gimbrone and García-Cardeña, 2016; Dei Cas et al., 2017; López et al., 2020), thus indicating an important role of circulating EPCs for restoring the integrity of the vascular endothelium in hypertension (Luo et al., 2016). Enhanced external counterpulsation (EECP) is a noninvasive modality for the treatment of ischemic cardiovascular disease. EECP therapy is done by sequential inflation of three sets of cuffs wrapped around the lower extremities during diastole and deflation of the cuffs during systole. EECP enhances the aortic diastolic blood flow and coronary perfusion, leading to increased arterial wall shear stress in a pulsatile manner (Luo et al., 2012; Yang and Wu, 2013; Raza et al., 2017). However, less is focused on EECP to regulate the function of EPCs in hypertension.

This study proposed to investigate the effect of EECP on BP and flow-mediated endothelial dilation (FMD). Furthermore, the correlation of EPCs capacities and FMD were examined to explore the underlying mechanism of EECP effect.

## Materials and Methods

### Study Population

This study was a prospective, randomized controlled study approved by the Medical Ethics Committee of Eighth Affiliated Hospital of Sun Yat-sen University. Forty outpatients aged 20–45 years with essential grade 1 hypertension without any medication treatment were included and randomized into the EECP group (*n* = 20) or control group (*n* = 20). Patients with essential grade 2 or 3 secondary hypertension, coronary artery disease (CAD), aortic valve disease, or medication history were excluded.

Ambulatory blood pressure monitering (ABPM) measurement was in accordance with the method recommended by the clinical guidelines (Whelton et al., 2018; Unger et al., 2020). Diagnosis of hypertension was based on three office measures of BP ≥ 140/90 mmHg at intervals no longer than 2 weeks, and 24-h mean ambulatory BP > 130/80 mmHg. ABPM was performed in all subjects before intervention and 1 day after intervention, by using an automatic portable device (Spacelab 90207, Redmond, WA, United States). Measurement was made every 30 min during 9:00 AM to 8:59 PM and every 60 min during 9:00 PM to 8:59 AM the next day.

FMD reflects artery endothelial dilation and is a marker of endothelial function (Lambiase et al., 2014). All subjects were assigned to perform FMD (Omron, UNEX EF 38G, Japan) examination before and 1 day after intervention. Greater than 9% was identified as normal.

### EECP Protocol

Complete EECP protocol was performed on all subjects in the EECP group with 35 times, 5 times a week (7 weeks in total), and 60 min for each time.

EECP therapy (PSK P-EECP/TI, Chongqing, China) consisted of a treatment bed attached to an air compressor unit that was attached to a computerized control console. The therapy used a series of three cuffs placed on the calves, lower thighs, and upper thighs/buttocks. The cuffs received sequential distal-to-proximal pneumatic inflation upon onset of diastole and simultaneous release of pressure at end-diastole. Sequential cuff inflation during EECP treatment produced two opposite blood flow patterns: antegrade flow in the brachial artery and retrograde flow in the femoral artery. Sequential cuff deflation also produced systolic hyperemia. The pressure applied to the cuffs was set at 0.035–0.040 mPa/cm^2^. Effective hemodynamic changes of EECP were demonstrated by achieving a diastolic-to-systolic ratio of 1.2 with the use of the plethysmographic technique (Yang and Wu, 2013).

### EPCs Culture and Characterization

EPCs were cultured and characterized as detailed previously (Zhang et al., 2014). Briefly, peripheral blood mononuclear cells from hypertensive subjects were cultured on fibronectin-coated six-well plates in endothelial cell basal medium-2 (EBM-2; Clonetics, San Diego, CA, United States) supplemented with endothelial growth medium-SingleQuots (contents: ascorbic acid 0.5 mL; rhFGF-B 2.0ML; heparin 0.5 mL; GA-1000 0.5 mL; rhEGF 0.5 mL; hydrocortisone 0.2 mL; VEGF 0.5 mL; R3-IGF-1 0.5 mL). Early EPCs were defined as cells dually positive for DiI-acLDL (0.02 mg/mL; Invitrogen, Carlsbad, CA, United States) uptake and FITC-labeled BS-1 lectin (0.01 mg/mL; Sigma-Aldrich, St. Louis, MO, United States) binding as previously described (Xia et al., 2017). Endothelial marker proteins of cultured EPCs were also examined by flow cytometry analysis by using phycoerythrin–labeled monoclonal mouse anti-human antibodies recognizing CD31 (BD Pharmingen), von Willebrand factor (vWF) (BD Pharmingen), and kinase-insert domain receptor (KDR) (R&D System). Furthermore, expression of the monocytic lineage marker CD14 (BD Pharmingen) was analyzed as previously described. Based on the isolation and cultivation protocol, the adherent mononuclear cells were identified as early EPCs.

### EPCs Proliferation, Migration, Adhesion, and Tube Formation Activities *in vitro*

The EPCs were incubated in a 96-well culture plate at 5 × 10^3^ cells/well. After incubation for 24 and 48 h, the cell proliferation was determined by Cell Counting Kit-8 (CCK-8) (Dojindo) according to the manufacturer’s protocol. The absorbance was measured at 450 nm using Elx800 Reader (Bio-Tek Instruments Inc., VT, United States). EPC migration was determined using a modified Boyden chamber. Briefly, 2 × 10^4^ EPCs, resuspended in 250 μL EBM-2, were pipetted in the upper chamber of a modified Boyden chamber (Costar Transwell^®^ assay, 8-μm pore size, Corning, NY, United States). The chamber was placed in a 24-well culture dish containing 500 μL EBM-2 supplemented with either phosphate-buffered saline (PBS) or 100 ng/mL SDF-1 (Peprotech, Rocky Hill, NJ, United States). After 24-h incubation at 37°C, transmigrated cells were counted by independent investigators blinded to grouping.

A monolayer of HUVECs was prepared 48 h before the assay by plating 2 × 105 cells in each well of a four-well plate. Human umbilical vein endothelial cells (HUVECs) were pretreated with or without 1 ng/mL tumor necrosis factor-α (Peprotech) for 12 h. Then 1 × 10^5^ CM-DiI (CellTracker^TM^ CM-DiI, Invitrogen)–labeled EPCs were added to each well and incubated for 3 h at 37°C. Nonattached cells were gently removed with PBS, and adherent EPCs were fixed with 4% paraformaldehyde and counted by independent investigators blinded to grouping.

EPC tube formation experiment was conducted as follows: a growth factor–reduced Matrigel (Corning) was warmed up at 4°C overnight. After having been completely thawed, 60 μL of Matrigel was plated to 96-well plates at the same level to distribute evenly, and incubated for 1 h at 37°C. Late EPCs (2 × 10^4^) were resuspended with EBM-2 and loaded on the top of the Matrigel. Each conditional group contained three wells. Following incubation at 37°C for 2 h, each well was imaged directly under a microscope, and an average of tubules was counted from three to five random fields.

### Statistical Analyses

All the results are expressed as mean ± standard deviation. The Student *t* test or Mann-Whitney unpaired test was used for comparison between groups. Correlations between continuous variables were assessed by Pearson correlation coefficient, and multivariate analysis was performed using multiple linear regression analysis. All *P* values were two-sided. Statistical difference was defined as *P* < 0.05 for all tests. Statistical analyses were performed using the Statistical Package for the Social Science (SPSS) software (version 22.0) (SPSS Inc, Chicago, IL, United States).

## Results

### Clinical Characteristics in EECP and Control Group at Baseline

We recruited 40 grade 1 hypertensive outpatients and randomized them into EECP [10 male and 10 female, mean age: (35.4 ± 5.6) years old] or control [11 male and 9 female, mean age: (34.8 ± 6.3) years old] group. The basic clinical characteristics of both groups are presented in Table 1. We observed no significant differences at baseline between the two groups.

**TABLE 1:** Demographic characteristics of both groups at baseline and end of intervention.

	**Baseline**		**After intervention**		**Before *t*/*χ*^2^**	**Before *P***	**After *t*/*χ*^2^**	**After *P***
	**EECP**	**Control**	**EECP**	**Control**				
*n*	20	20	20	20				
Age (years)	35.4 ± 5.6	34.8 ± 6.3			1.12	0.38		
Male (%)	10/50	11/55			0.021	0.88		
Smoking (%)	12/60	9/45			0.45	0.56		
Hypertension (%)	11/55	10/50			1.13	0.29		
Diabetes (%)	2/10	1/5			0.41	0.52		
Mean SBP (mmHg)	139.3 ± 6.4	137.4 ± 5.8	133.2 ± 4.9^a^	136.9 ± 6.2	0.12	0.73	2.12	0.08
Mean DBP (mmHg)	89.5 ± 7.6	88.6 ± 8.10	83.4 ± 4.5^a^	87.3 ± 5.5	0.13	0.72	1.45	0.04
HR (bpm)	74	71	69	70	0.06	0.88	1.02	0.57
FMD (%)	7.21 ± 2.32	7.56 ± 1.72	8.87 ± 2.46^a^	7.88 ± 1.55	1.28	0.26	1.92	0.10
CRP (mg/L)	1.8 ± 1.1	2.0 ± 1.06	1.6 ± 0.45	1.89 ± 0.68	–0.78	0.53	–1.12	0.11
Cr (μmol/L)	73.97 ± 17.63	71.85 ± 12.87	70.93 ± 18.69	67.64 ± 8.40	–0.374	0.096	0.604	0.55
LDL (mmol/L)	3.22 ± 0.44	3.04 ± 0.49	2.82 ± 0.69	2.78 ± 0.23	1.3	0.65	1.12	0.69
FBG (mmol/L)	5.0 ± 1.2	4.8 ± 0.98	4.8 ± 1.1	4.9 ± 0.83	0.54	0.49	0.31	0.57

*^*a*^There is significant difference before and after EECP; *P* < 0.05.*

*FMD, flow-mediated endothelial dilation; CRP, C-reactive protein; Cr, serum creatinine; LDL, low density lipoprotein; FBG, fasting blood glucose.*

### Effect of EECP on BP and FMD in Both Groups

**FIGURE 1:**
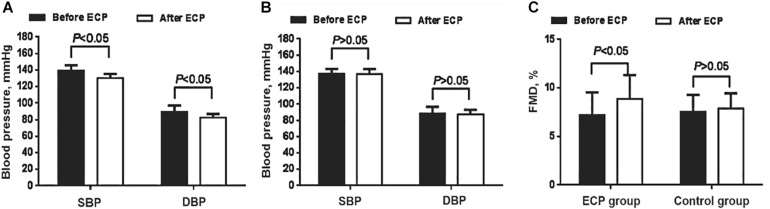
Comparison of blood pressure and FMD change in both groups. **(A)** Both SBP and DBP decreased after EECP in the interventional group (*P* < 0.05). **(B)** Neither SBP nor DBP decreased in the control group. **(C)** FMD increased after EECP in the interventional group (*P* < 0.05); no similar pattern was found in the control group. EECP, enhanced external counterpulsation; SBP, systolic blood pressure; DBP, diastolic blood pressure; FMD, flow-mediated endothelial dilation.

ABPM and office BP are the standard methods for the diagnosis of hypertension. Both systolic [SBP: (133.2 ± 4.9) mmHg vs. (139.3 ± 6.4) mmHg, *P* < 0.05] and diastolic BP [DBP: (83.4 ± 4.5) mmHg vs. (89.5 ± 7.6) mmHg, *P* < 0.05] of EECP group decreased greatly, whereas there was no obvious difference in the control (Table 1 and Figures 1A,B). FMD reflects the endothelial dilation of artery and our results showed that FMD of EECP group increased significantly than that of the control group [(8.87 ± 2.46%) vs. (7.51 ± 2.32%), *P* < 0.01]. However, no significant change (*P* > 0.05) was observed in the control group (Table 1 and Figure 1C).

### Characterization of Early EPCs

**FIGURE 2:**
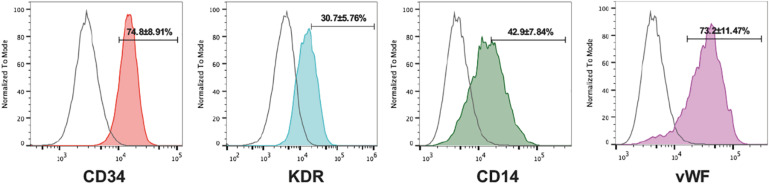
Characterization of cultured EPCs. Flow cytometry analysis of the endothelial markers CD31, vWF, KDR, and the monocytic lineage marker CD14 of EPCs (IgG isotype control shown in red, *n* = 5 per group). Numbers are the mean ± SEM percentage of positive cells for all experiments determined by comparison with corresponding negative control labeling.

We grew early EPCs by culturing PBMCs in specific media for 7 days. Fluorescent staining showed that the vast majority of adherent cells expressed endothelial marker proteins (CD31, vWF, and KDR) and a monocytic marker CD14 at comparable levels (Figure 2). All the characterizations above indicated that the cultured EPCs in this study could be classified into early outgrowth EPCs as descripted previously (Zhang et al., 2014).

### EECP Restored the Proliferation, Migration, Adhesion, and Tube Formation Capacity of EPCs in Hypertension

**FIGURE 3:**
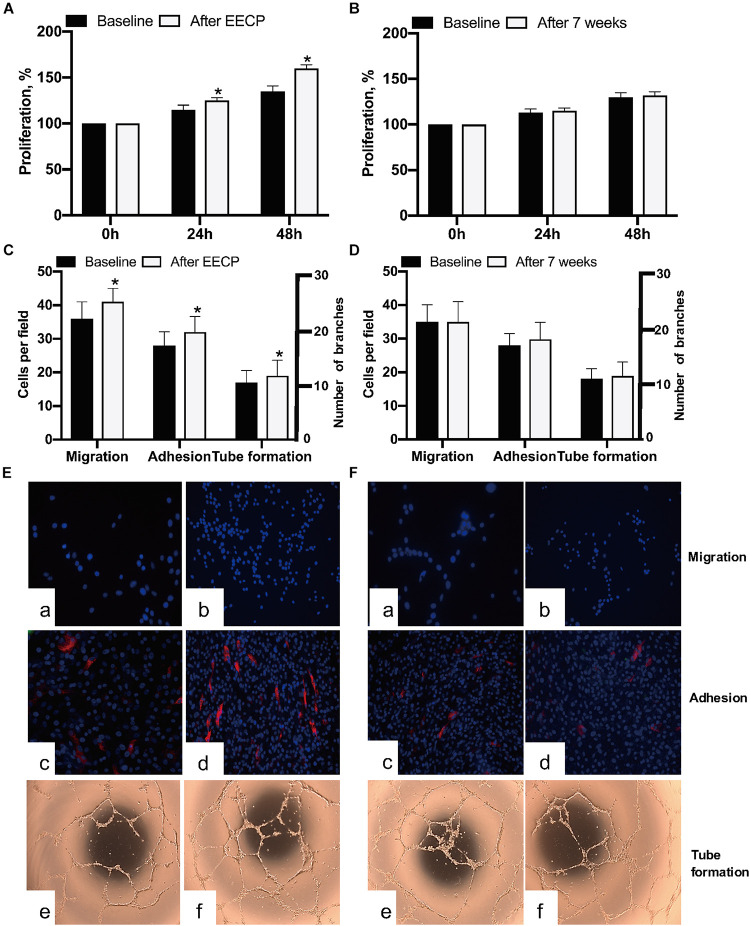
Comparison of EPCs activities in both groups before and after EECP. **(A,B)** Effect of EECP on EPCs proliferation. **(C,D)** Quantification analyses elucidated the differences of migration, adhesion, and tube formation capacity in EECP and control group. **(E)** Representative photographs showing EECP improved migration **(a,b)**, adhesion **(c,d)**, and tube formation **(e,f)** of EPCs in the interventional group. **(F)** Representative photographs showing no changes of migration **(a,b)**, adhesion **(c,d)**, and tube formation **(e,f)** found in the control group after 7 weeks. * means the difference between groups is statistically significant (*P* < 0.05). EECP: enhanced external counterpulsation; EPCs: endothelial progenitor cells.

After 7-day cultivation, EPCs were successfully obtained and performed proliferation, migration, and adhesion activities examinations; after 28-day cultivation, tube formation was performed accordingly. Our data indicated EECP intervention reinforced the proliferation (*P* < 0.05), migration (*P* < 0.05), adhesion (*P* < 0.05), and tube formation (*P* < 0.05) capacities of EPCs. However, no similar pattern was found in the control (Figure 3).

### EPCs Activities Correlated With FMD in Hypertension

**FIGURE 4:**
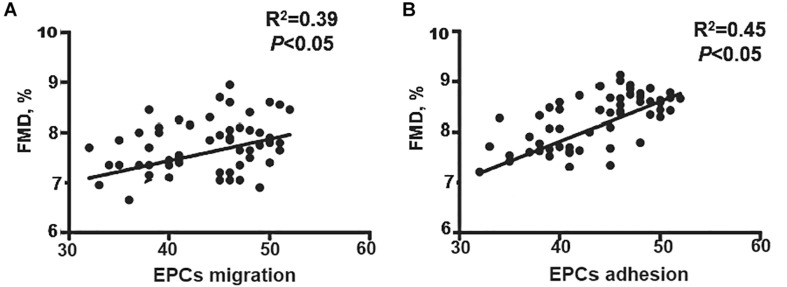
Correlation analysis of EPCs activities and FMD. **(A)** EPC migration positively correlated with FMD. **(B)** EPCs adhesion positively correlated with FMD. EECP, enhanced external counterpulsation; EPCs, endothelial progenitor cells; FMD, flow-mediated endothelial dilation; CR, C-reactive protein; Cr, serum creatinine; LDL, low density lipoprotein; FBG, fasting blood glucose.

Although the results show that EECP increased FMD and improved EPC function, the correlation between FMD and EPCs capacities remained unknown. Migration and adhesion capacity was correlated with flow-mediated diastolic function, so we analyzed the correlation between FMD and migration–adhesion capacity of EPCs. Our results showed that both the migration [odds ratio (OR) = 0.47, 95% confidence interval (CI) = 0.27–0.64, *P* < 0.05) and adhesion (OR = 0.44, 95% CI = −0.0034 to 0.0012, *P* < 0.05) function of EPCs correlated with FMD (Figures 4A,B). After multivariate analysis, we found the migration (*β* = 3.37, 95% CI = 1.67–5.33, *P* < 0.05) and adhesion (*β* = 3.98, 95% CI = 1.12–6.43, *P* < 0.05) activities were still associated with FMD, which indicated that the migration and adhesion functions were independent predictors for FMD decline (Table 2).

**TABLE 2:** Multivariate logistic analysis for the correlation of FMD and relative factors.

	***β* value**	***P* value**	**OR**
EPC migration	3.37	0.03	28.32
EPC adhesion	3.98	0.04	25.4
CRP	4.21	0.08	16.10
FBG	1.34	0.21	9.54
Cr	0.34	0.80	1.41
LDL	–0.94	0.54	0.82
Age	0.18	0.11	6.84

## Discussion

The present study revealed that (1) EECP reduced both SBP and DBP in hypertension; (2) EECP increased endothelium-dependent, flow-mediated vasodilation; (3) declined EPC capacity in patients with hypertension could be restored by EECP and independently correlated with FMD. Our findings for the first time provide novel evidence that EECP regulates EPC capacity to repair hypertensive vascular endothelial injury, and EECP may become a noninvasive approach for hypertension management besides medication.

The impact of hypertension for the increased risk of cardiovascular disease is initiated by altering endothelial function of the arterial vessels. Endothelium plays a critical role in regulating arterial compliance and resistance. Endothelial cells release numerous vasodilator and vasoconstrictor substances that help regulate blood flow and vascular tone during rest and exercise (Xia et al., 2012). FMD of the brachial artery, assessed noninvasively via ultrasound, has been validated in youth as a marker of vascular endothelial function (Lambiase et al., 2014). Our data elucidated that FMD index (7.1 ± 2.1%) was relatively lower than the normal 9% in the early stage of hypertension. Thus, it is of great significance to explore effective means not only to lower BP but also to maintain endothelial integrity.

EECP is a noninvasive therapy for the treatment of patients with CAD. Growing evidence suggests that improvement in endothelial function represents an important mechanism for the clinical benefits of EECP (Luo et al., 2012; Yang and Wu, 2013). Acute increase in shear stress increases blood flow and causes acute robust nitric oxide (NO) production, which plays a critical role in vessel relaxation. Chronic NO production due to the increased laminal shear stress may serve as an antiatherogenic and anti-inflammatory molecule, which in turn enhances the endothelial NO synthase/NO pathway (Green et al., 2017). NO-mediated vasodilation is an important way to lower DBP, resulting in decreased peripheral vascular resistance, which further lowers SBP. The previous study indicated the EECP increases the central aorta pressure and decreases the systolic pressure; in addition, elevated intra-arterial pressure was found during EECP (Michaels et al., 2002; Beck et al., 2015). Picard (Picard et al., 2020) found individual shear rate therapy; further development of EECP decreased both SBP and DBP in patients with CAD. However, there was also another study that demonstrated EECP has no lasting effect on ambulatory BP (May and Khair, 2013). The inconsistent results need to be further analyzed. First, the subjects enrolled in the previous studies had CAD, stroke, or left ventricular dysfunction; different disease statement results from different vascular and myocardial pathological change; and results in different reaction to EECP effect. Second, most subjects enrolled in the studies took different antihypertensive medication and may interfere the effect of EECP. In the recent study, the enrolled subjects were relatively young and with mild hypertension without any antihypertensive medication; the results directly reflected the effect of EECP on peripheral BP. We demonstrated that EECP effectively decreased both SBP and DBP in the mild hypertensive patients without any antihypertensive medication. In addition, EECP increased FMD in hypertension, indicating that the BP-lowering effect of EECP may partially contribute to regulating endothelial dependent vasodilation. However, the underlying mechanism of EECP in regulating endothelial function in hypertension has not been completely defined.

Imbalance between endothelial injury and repair requires a more effective approach to maintain endothelial homeostasis. Thus, regeneration of the vascular endothelium is of great importance (Goligorsky, 2014; Liu et al., 2018). The endothelial repair can occur by migration and proliferation of surrounding mature ECs. However, mature endothelial cells are terminally differentiated cells with low proliferative potential, and their capacity to substitute damaged endothelium is limited. Accumulating evidence indicates that circulating EPCs may contribute to ongoing endothelial repair, and the level of circulating EPCs is closely correlated with endothelial function (Burlacu et al., 2013; Hirschi and Dejana, 2018). Our previous studies revealed that EECP could restore endothelial function by promoting NO release, counteracting inflammation and inhibiting the proliferation and migration of vascular smooth muscle cells (Eman et al., 2014; Bianconi et al., 2018). Whether EECP can improve EPC function and relationship between EPC function and arterial FMD under EECP treatment remains unknown.

In the present study, we found both EPC migration and adhesion activities positively correlated with FMD. After multivariate analysis, the correlation still existed. Therefore, EPC function can be considered as the independent predictor of endothelial-dependent vasodilation. Our results validated that endothelial-mediated vasodilation independently correlated to EPC function, which could be restored by EECP in hypertension. Collectively, EECP can improve endothelial-dependent vasodilation and lower BP in hypertension via regulating EPC function.

## Potential Study Limitations

It should be pointed out that this study has some limitations. First, this is a small sample size study; a larger-population study should be performed to strengthen these results. Second, the exact molecular mechanisms underlying the impairment of EPC functions related to vascular injury should be further elucidated in the future. Third, the efficacy of EECP on BP in different grades and stage of hypertension should be quantitatively analyzed by further prospective randomized clinical trials.

## Conclusion

In summary, we demonstrate for the first time that EECP modulates EPC function to improve endothelial-related vascular dilation. Taken the effect of EECP on BP lowering together, our data provide an impetus for future evaluation of EECP in hypertension therapeutic efficacy and prognosis.

## Data Availability Statement

The raw data supporting the conclusions of this article will be made available by the authors, without undue reservation.

## Ethics Statement

The studies involving human participants were reviewed and approved by the Medical Ethics Committee of Eighth Affiliated Hospital of Sun Yat-sen University. The patients/participants provided their written informed consent to participate in this study.

## Author Contributions

GW designed the project and are responsible for the overall content, contributed to revise the manuscript. JL, JS, and WW carried out all the experiments. JL and JS prepared the manuscript. All authors have seen and approved the final manuscript.

## Conflict of Interest

The authors declare that the research was conducted in the absence of any commercial or financial relationships that could be construed as a potential conflict of interest.
